# Insights into Intraspecies Variation in Primate Prosocial Behavior: Capuchins (*Cebus apella*) Fail to Show Prosociality on a Touchscreen Task

**DOI:** 10.3390/bs4020087

**Published:** 2014-04-10

**Authors:** Lindsey A. Drayton, Laurie R. Santos

**Affiliations:** Department of Psychology, Yale University, 2 Hillhouse Avenue, New Haven, CT 06511, USA; E-Mail: lindsey.drayton@gmail.com

**Keywords:** prosociality, altruism, capuchins, non-human primates, donation tasks

## Abstract

Over the past decade, many researchers have used food donation tasks to test whether nonhuman primates show human-like patterns of prosocial behavior in experimental settings. Although these tasks are elegant in their simplicity, performance within and across species is difficult to explain under a unified theoretical framework. Here, we attempt to better understand variation in prosociality by examining the circumstances that promote and hinder the expression of prosocial preferences. To this end, we tested whether capuchin monkeys (*Cebus apella*)—a species that has previously demonstrated prosocial preferences—would behave prosocially using a novel touchscreen task. In contrast to previous studies, we found that capuchins as a group did not prosocially deliver food to a partner. Importantly however, data from control conditions revealed that subjects demonstrated limited understanding of the reward contingencies of the task. We also compared individuals’ performance in the current study with their performance in a previously published prosociality study. We conclude by discussing how continuing to explore intraspecies variation in performance on prosocial tasks may help inform debates regarding the existence of other-regarding preferences in nonhuman species.

## 1. Introduction

Although humans may be unique among species in the extent to which we help others, there can little doubt that non-human primates also act in ways that benefit other individuals in some contexts. Some primate species are known to transfer food to unrelated social partners [1], engage in instrumental helping [2,3,4,5,6], and console victims of aggression [7]. Such behaviors are often referred to as *prosocial behaviors.* Note that unlike the term altruism, the term prosociality does not make any stipulations regarding the cost of the behavior to the actor. The extent to which other species share human-like prosocial motivations is controversial, but understanding interspecies variation in prosocial tendencies is critical to furthering our understanding of the evolutionary origins of human altruism.

Recently, there has been renewed interest in the use of experimental paradigms to test whether different primate species exhibit prosocial behaviors (for a review, see Silk and House [8]). In a widely used paradigm, one individual (the “actor”) must choose between two different options. Although both options yield the same food reward for the actor, one option delivers a highly desired food to a partner (the “recipient”) while the other option does not. Consequently, the actor must choose whether or not to deliver food to the recipient at no (or very minimal) personal cost. The rationale of the design is that if actors are *indifferent* to the welfare of the recipient then they should choose randomly between the two options. If actors are *antisocial* (*i.e*., they have a preference for decreasing other individuals’ welfare), then they should choose the less good option more often when the recipient is present than in a control condition in which no recipient is present to retrieve the food reward. Finally, if actors are *prosocial*, then they should choose the generous option more often when a recipient is present to retrieve the food reward than in control conditions in which no recipient is present.

To date, many researchers have capitalized on this simple design to test primates’ prosocial preferences [9,10,11,12,13,14,15,16,17,18,19,20,21,22]. Unfortunately, these experiments together have produced a rather puzzling set of results. For example, species may demonstrate prosocial behavior in some studies but not others (e.g., chimpanzees [9,13,14,18,21]; tamarins [10,11,19]). Additionally, even when there is an overall difference in performance between recipient present and recipient absent conditions, prosocial behavior does not necessarily manifest in all actor-recipient dyads (e.g., marmosets [22]; capuchins [12,15,20]). Whether such differences are the product of variation in individuals’ motivation to engage in prosocial behaviors per se or instead reflect differences in task understanding, sensitivity to inequity, attentional biases, or quality of relationship with the recipient is currently unclear. Finally, the overall percent differences between the recipient present and recipient absent conditions are often fairly modest [19]. These results raise questions regarding the degree to which prosociality is affected by particular features of the testing environment, as well as the extent to which prosocial behaviors, when observed, can be described as species typical or consistent with a robust preference to help others. Given these open questions, it has been difficult to explain within and across species performance within a single theoretical framework.

To help address these issues it may be useful to further explore the stability of prosocial behaviors, both at the species and individual level. To do this, we decided to test brown capuchin monkeys using a novel testing method. Capuchin monkeys are appropriate for assessing the stability of prosocial preferences for a number of reasons. This species exhibits high social tolerance and is able to successfully cooperate in a variety of tasks [23,24,25]. Moreover, the results of previous tests suggest that capuchins do act prosocially in a number of contexts [12,15,20]. Nevertheless, this same set of studies also provides evidence that prosociality in capuchins may ultimately be quite fragile and subject to many of the inconsistencies listed above. That is, prosocial behaviors do not necessarily characterize all individuals or dyads and mean differences between the test and control conditions are often quite small (e.g., approximately 8% in Lakshminarayanan and Santos [15]). Capuchins’ prosocial preferences may also disappear when the proposer has limited visual access to the recipient [12,20]. Indeed, in such cases capuchins sometimes actually behave antisocially, preferring the antisocial option to a prosocial one [12]. Finally, at least one study [20] has reported that prosocial choices were directed primarily down the dominance hierarchy, but this pattern has not been confirmed in other studies [12].

In order to further our understanding of the conditions under which capuchins exhibit prosocial behavior, we tested subjects using a novel touchscreen task. Computer-based tasks have proven useful in exploring primate cognition across a number of domains (e.g., memory [26]; metacognition [27]; gaze following [28]; social decision-making [29]). As in previous studies, monkeys could choose between a prosocial option (food for the recipient) and a selfish option (no food for the recipient). Three of the actors in this study had previously participated in another study exploring prosocial preferences [15]. Note that in this previous study, capuchins as a group demonstrated prosocial behavior. That is, they selected the prosocial option more often when a recipient monkey was in the adjacent testing chamber compared to when no recipient was present. Because we were particularly interested in whether these three subjects would exhibit a similar pattern of preferences in two different contexts, we analyzed each actor’s performance in the two studies using the same statistical tests, thus allowing us to begin to explore performance across different contexts and different testing methods. Importantly, in addition to including a control condition in which no recipient was present to receive food rewards, we also included an additional control condition designed to probe subjects’ understanding of the contingencies of their responses. This is critical because the difficulties inherent to understanding inter and intra-species inconsistencies in performance across various studies are exacerbated by the fact that it is not always clear what subjects understand about the reward contingencies of their responses on specific tasks. Specifically, if subjects show an indifferent pattern of responding (that is, selecting the prosocial option equally often when a recipient is present compared to when the recipient is absent), it can be difficult to know whether this is because subjects are in fact indifferent to their partner’s payoff or whether subjects are simply not attending to all the relevant features of the task.

## 2. Method

### 2.1. Subjects

Five brown capuchin monkeys living in a single social group at the Comparative Cognition Laboratory at Yale University participated in this study. Two adult males (FL and NN), one adult female (JM), and one adolescent female (MP) acted as actors. A low-ranking female (HR) acted as the recipient in all testing pairs. We chose a low-ranking recipient because previous research suggests that capuchins may be more likely to behave prosocially towards a low-ranking partner than towards a high-ranking partner [20]. We also chose to limit relatedness within the dyads; although FL was the likely father of HR, all other actor-recipient dyads were unrelated even though they had lived together from birth. All subjects had participated in a wide range of cognitive experiments, and three of the actor monkeys (FL, NN, and JM) had participated in a previous study exploring capuchins’ prosocial preferences [15]. Additionally, all of the actors had been trained to interact with a touchscreen for previous studies (e.g., Furlong and Santos [30]) and, thus were skilled at selecting stimuli presented on the screen.

Monkeys were fed twice daily on a diet of monkey chow, fruits, vegetables, and other snack items and had ad libitum access to water. Participation in this study was entirely voluntary. Although monkeys were physically isolated during all testing sessions, they always had visual and auditory access to other group members.

### 2.2. Testing Apparatus

**Figure 1:**
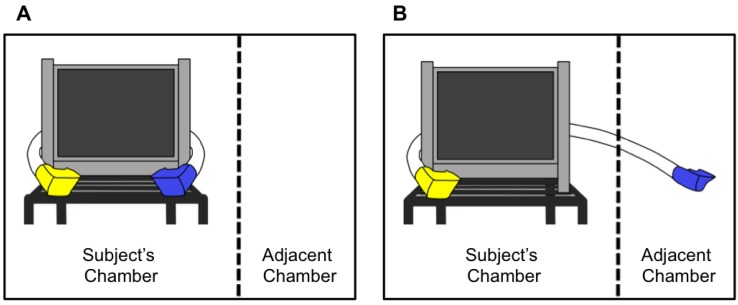
The schematic on the left (**A**) illustrates the testing setup in conditions in which the actor had access to both the cup on the right and the cup on the left (*i.e*., *selfish training sessions* and *selfish control sessions*). The adjacent chamber was always empty. The schematic on the right (**B**) illustrates the testing setup in conditions in which the actor had access to only the cup on the left. The adjacent chamber was either empty (*i.e.*, *empty training sessions* and *empty control sessions*) or contained the recipient monkey (*i.e*., *prosocial test sessions*).

The testing apparatus consisted of a 15-inch touchscreen monitor on a mobile cart (Figure 1). Transparent tubes (1.5 inch diameter) were attached to the left and right sides of the testing apparatus, allowing the experimenter to deliver food rewards (Kix brand breakfast cereal) to the actor and/or recipient monkey from behind the cart. Food rewards were delivered into a yellow cup that was placed at the end of one tube and a blue cup that was placed at the end of the other tube. The actor always had access to rewards in the yellow cup; however, depending upon the condition (see Procedure section), the actor, the receiver, or no one had access to the rewards in the blue cup. In conditions in which the actor had access to both cups, the two cups were attached to the front of the cart within reaching distance of the actor (Figure 1A). In conditions in which the actor had access to the yellow cup only, the yellow cup was attached to the front of the cart but the blue cup was only accessible through the adjacent testing chamber (Figure 1B), which could either be empty or contain the recipient monkey. At the beginning of each session, the cart was placed in front of the actor monkey, allowing him or her to interact with the touchscreen through a 13.5 × 15 inch testing window.

### 2.3. Stimuli

**Figure 2:**
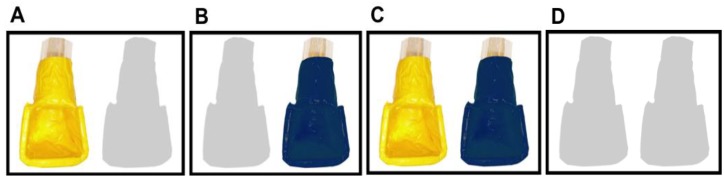
Images of the four stimuli representing four different food reward outcomes: (**A**) 1/0; (**B**) 0/1; (**C**) 1/1; (**D**) 0/0.

All stimuli were presented on the touchscreen using a computer running SuperLab 4.5 software. Four different stimuli were used (see Figure 2), each of which represented a different reward outcome. The first option delivered food only to the yellow cup (1/0). The second delivered food only to the blue cup (0/1). The third delivered food to both cups (1/1), and the forth delivered food to neither cup (0/0). Following previous studies (e.g., Silk *et al.* [8]) we refer to these throughout the paper as 1/0, 0/1, 1/1, 0/0 throughout the paper. Note that in this nomenclature, the first number represents whether a food reward was delivered to the yellow cup while the second number represents whether a food reward was delivered to the blue cup.

### 2.4. Procedure

#### 2.4.1. Stimulus Preference Control Session

We first wanted to verify that monkeys did not have a bias towards any of the stimuli based on perceptual features alone. Each actor therefore participated in a pilot study consisting of a single session of 16 single option trials, in which only one stimulus was presented (each four times), followed by 36 choice trials, in which two stimuli were presented simultaneously. At the onset of each choice trial, a black fixation cross was presented on the screen. This was followed by the presentation of the two stimuli. Each monkey experienced six possible pairs of stimuli, and each pair was presented a total of six times. Stimuli were displayed at the center-top and center-bottom of the touchscreen. Once the actor made a choice, the selected stimulus became larger and changed slightly in appearance, and the other stimulus disappeared. This change was accompanied by a sound. The actor always received a single food reward upon making a selection, which was delivered through a polyvinyl chloride (PVC) tube. The dual-tube delivery system was not present.

#### 2.4.2. Training on Reward Contingencies

Following pilot testing, actor monkeys participated in a series of 16 training sessions to familiarize them with the testing apparatus, tubes, and outcomes of the stimuli. Subjects were physically separated from other group members in one of two side-by-side testing chambers (1 m × 1.6 m × 2.4 m) directly adjacent to the monkeys’ home enclosure. Mesh caging and a Lexan panel separated the two testing compartments. During all training sessions the second testing chamber was empty. Each training session consisted of 32 trials in which only one stimulus was available. As in the pilot study, a fixation was displayed on the screen at the onset of each trial; however, this was followed by the presentation of only one of the four stimuli. Each stimulus was presented a total of eight times per sessions and the location of the stimulus on the touchscreen was counterbalanced. The order in which the stimuli were presented was determined randomly.

We used two different types of training sessions: *selfish training sessions* and *empty training sessions*. In *selfish training sessions*, actors were able to retrieve food from both the yellow and the blue cup (as in Figure 1A). In *empty training sessions*, actors were only able to retrieve food from the yellow cup (as in Figure 1B). Because during *empty training sessions* the blue cup was accessible only from inside the empty testing chamber, food rewards delivered to the blue cup simply remained inside the cup until the end of the training sessions, at which point the experimenter removed them. Training sessions alternated between the two conditions.

#### 2.4.3. Test Sessions

After completing training, actors participated in three sets of three testing sessions. Each set consisted of a one *prosocial test session*, one *empty control session*, and one *selfish control session*, for a total of nine testing sessions per actor. The order in which the different sessions were presented within each set was partially counterbalanced such that each type of session was presented to at least one actor first and to at least one actor last. In *prosocial test sessions*, the actor monkey had access to the yellow cup only. The recipient monkey (HR) was in the second testing chamber and could retrieve food rewards delivered to the blue cup through a 13.5 × 15 inch testing window. The actor monkey had visual and auditory access to the receiver and could clearly see the recipient retrieving food rewards from the blue cup. In order to test whether actors’ choices were dependent upon the presence of a conspecific, *empty control sessions* were included in which the actor again had access to only the yellow cup, but no recipient monkey was present in the second testing chamber. Finally, *selfish control sessions* were included to gauge actors’ understanding of the task and the various outcomes represented by the stimuli. In these sessions, actors had access to food rewards in both the yellow and the blue cup. Note that physical features of the test environment (*i.e*., access to cups, presence/absence of recipient) in *selfish control sessions* and *empty control sessions* were identical to those in *selfish training sessions* and *empty training sessions*, respectively. In addition, in all conditions subjects always had visual and auditory access to other group members in the home enclosure. 

In each test session, actors first completed eight trials in which only one stimulus was present (two of each stimulus) to remind them of the reward outcomes of each stimulus. These single option trials were followed by 32 choice trials presented in four blocks. Each block consisted of eight presentations of a pair of stimuli. The four pairs of stimuli were: 1/1 *vs.* 1/0, 0/1 *vs.* 0/0, 1/0 *vs.* 0/0, and 0/1 *vs.* 1/1. The location of each stimulus was counterbalanced within each block, and the order of blocks was randomized within each session. Note that two pairs (1/1 *vs.* 1/0 and 0/1 *vs.* 0/0,) offered the choice between a prosocial option (1/1 and 0/1, respectively) and a selfish option (1/0 and 0/0, respectively). Thus, we predicted that for these two pairs of stimuli actors would choose the prosocial option more often in *prosocial test sessions* (when the receiver would be able to retrieve food rewards delivered to the blue cup) and *selfish control sessions* (when the actor would be able to retrieve food rewards delivered to the blue cup)than in *empty control sessions* (when neither the actor nor the receiver could retrieve food rewards delivered to the blue cup). The other two pairs of stimuli (1/0 *vs.* 0/0 and 0/1 *vs.* 1/1) offered an optimal selfish choice regardless of test condition (1/0 and 1/1, respectively). Thus, we predicted that for these pairs of stimuli actors’ choices would not differ across conditions.

The computer automatically recorded actors’ stimulus selections. Due to the small sample size involved in the study, nonparametric statistics were used. All tests are two-tailed.

## 3. Results

### 3.1. Stimulus Preference Control Sessions

We first verified that prior to training actors did not have a bias towards any of the stimuli based on perceptual features alone. To do this, we compared the number of times that each actor selected each stimulus in the *stimulus preference control session* to chance levels using binomial tests. Because four tests (one for each stimulus) were required for each actor, a Bonferroni adjusted alpha level of 0.0125 was used. No actor exhibited a bias towards any of the stimuli (FL: all *p*s > 0.23; JM: all *p*s > 0.48; MP: all *p*s > 0.09; NN: all *p*s > 0.99).

### 3.2. Test Sessions

**Figure 3:**
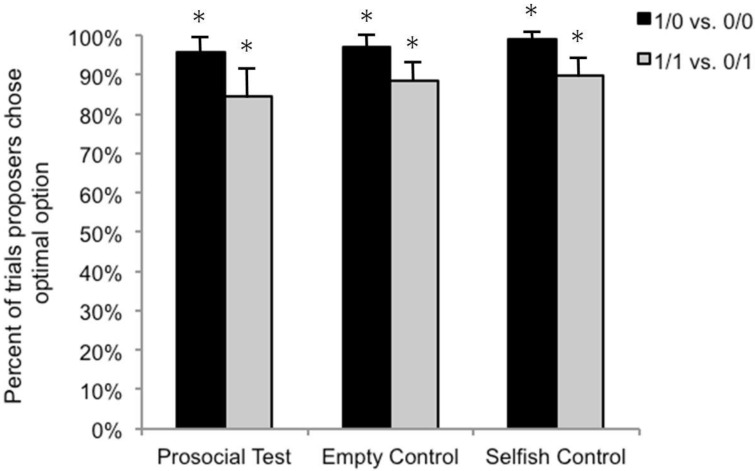
Mean percent (+SE) of optimal choices by actors in the three conditions. Asterisks denote when actors’ behavior was significantly different from chance.

We then analyzed performance in the test sessions. We first wanted to determine whether actors acted optimally and chose the stimulus that always provided them with a food reward when presented with the pairs of stimuli that always offered an optimal choice regardless of the condition, that is 1/0 *vs.* 0/0 (optimal choice: 1/0) and 1/1 *vs.* 0/1 (optimal choice: 1/1). Using binomial tests, we compared the number of times that actors as a group selected the optimal choice to chance levels and found that responses differed from chance for both stimulus pairs in each of the three conditions (*N* = 96 total observations across monkeys, all *p*s < 0.001; Figure 3). Collapsing across conditions, we found that actors chose the 1/0 option over the 0/0 option on 97% of all trials and that each individual actor chose the 1/0 option at greater than chance levels (all *p*s < 0.001). Similarly, when given the choice between 1/1 and 0/1, actors chose the 1/1 option on 88% of all trials and each individual actor chose the 1/1 option at greater than chance levels (all *p*s < 0.001). We then tested our hypothesis that there would be no difference in actors’ choices across the different conditions. Results of Friedman’s tests supported our prediction (1/0 *vs.* 0/0: χ^2^(2) = 2, *p*> 0.999; 1/1 *vs.* 0/1: χ^2^(2) = 0.667, *p*> 0.999), suggesting that when choosing rewards only for themselves, capuchins chose equally well across the different conditions.

**Figure 4:**
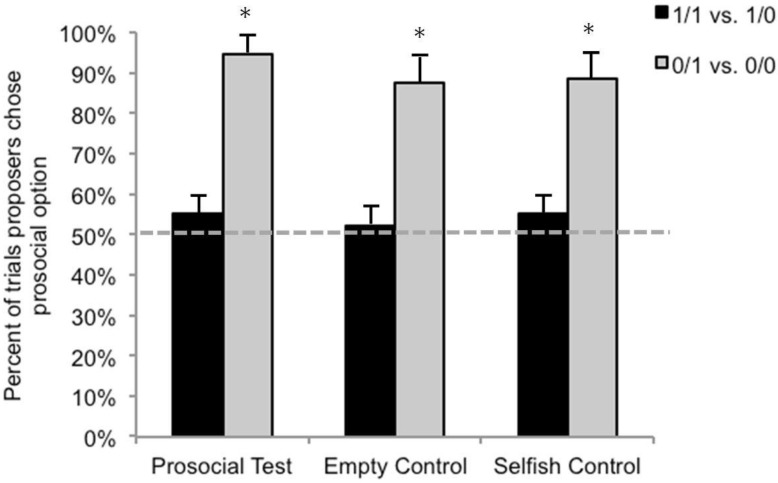
Mean percent (+SE) of prosocial choices by actors in the three conditions. Asterisks denote when actors’ behavior was significantly different from chance.

Turning to the two pairs of stimuli that offered a prosocial and a selfish option (1/1 *vs.* 1/0 and 0/1 *vs.* 0/0), we compared the number of times in each condition that actors as a group selected the prosocial stimulus (1/1 and 0/1, respectively) to chance levels, again using binomial tests. When offered the choice between 1/1 and 1/0, actors chose the prosocial option on 54% of all trials. In no condition did actors as a group choose the prosocial stimulus at levels significantly different from chance (*N* = 96 total observations across all monkeys, all *p*s > 0.35; Figure 4)*.* A Friedman’s test revealed that, contrary to our predictions, actors did not select the prosocial stimulus at different rates across the different test conditions (χ^2^(2) = 0.143, *p* = 0.944). Nevertheless, to test our directional hypotheses we conducted planned comparisons between *empty control sessions* and *selfish control sessions* and between the *empty control sessions* and *prosocial test sessions* using two-tailed Wilcoxon Signed-Rank tests with a Bonferroni adjusted alpha level of 0.025 per test. We found no difference in the group’s behavior in *empty control sessions* and *selfish control sessions* (*Z* = −0.736, *p* = 0.5) or in *empty control sessions* and *prosocial test sessions* (*Z* = 0, *p* > 0.999).

When offered the choice between 0/1 and 0/0, actors as a group chose the prosocial option on 90% of all trials and at greater than chance levels in every condition (*N* = 96 total observations collapsed across all monkeys, all *p*s < 0.001; Figure 4). However, once again the actors did not select the prosocial stimulus at different rates across the different conditions (χ^2^(2) = 3.8, *p* = 0.333). We followed up with planned comparisons and found no difference in the group’s behavior in *empty control sessions* and *selfish control sessions* (*Z* = 0, *p* > 0.999) or between *empty control sessions* and *prosocial test sessions* (*Z* = −1.633, *p* = 0.25).

**Figure 5:**
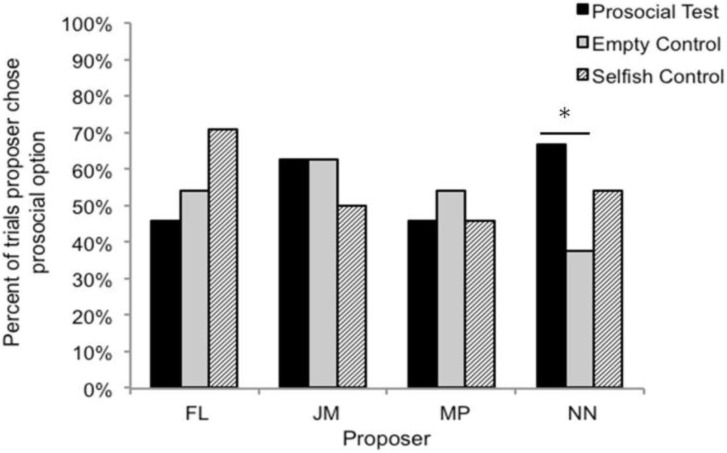
Percent of trials each actor selected the prosocial choice (1/1) when presented with the choice between 1/1 and 1/0 in the three conditions. Asterisks denote when an actor’s behavior was significantly different between conditions.

**Figure 6:**
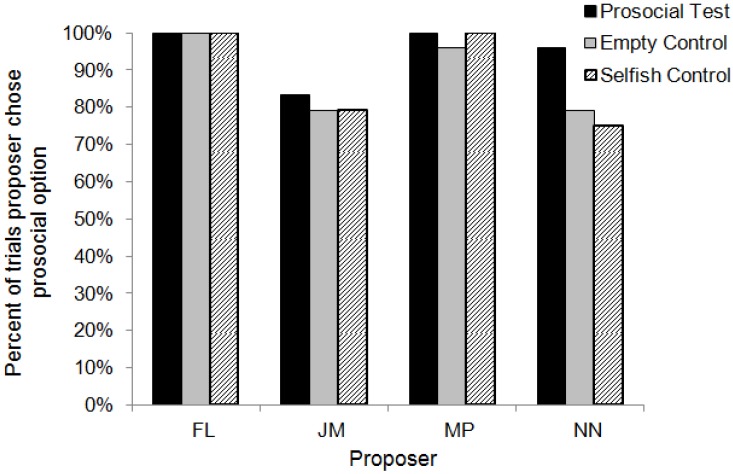
Percent of trials each actor selected the prosocial choice (0/1) when presented with the choice between 0/1 and 0/0 in the three conditions.

Although as a group actors did not exhibit different stimulus preferences across the three conditions, we also explored whether any individual actor might have done so (see Figure 5 and Figure 6 for data on individual actors). For each stimulus pair of interest (*i.e.*, 1/1 *vs.* 1/0 and 0/1 *vs.* 0/0) we conducted two additional planned comparisons per actor to test whether any individual actor might have chosen the prosocial option more often in either *selfish control sessions* or *prosocial test sessions* than would be expected based on the number of times he or she chose the prosocial stimulus in the *empty control condition*. Binomial tests were used with the probability of success on a single trial set to the percent of time that the actor had selected the prosocial stimulus across all *empty control sessions*. As in previous planned comparisons, we set an adjusted alpha level of 0.025 per test. Results of the planned comparisons revealed that one actor (NN) selected the 1/1 option over the 1/0 option more often in *prosocial test sessions* than in *empty control sessions* (*p* = 0.008) and also exhibited a trend towards selecting 0/1 over 0/0 more often in *prosocial test sessions* than in *empty control sessions* (*p* = 0.054). No other significant differences were found. 

### 3.3. Comparison with Lakshminarayanan and Santos (2008)

Using the same analyses described above (*i.e.*, two-tailed binomial tests), we tested whether FL, NN, and JM behaved prosocially in the previous Lakshminarayanan and Santos [15] study. (Note that MP’s data could not be analyzed since she was too young to be have previously been tested in the other prosociality studies). In Lakshminarayanan and Santos [15], actors were given the opportunity to deliver a high-value (prosocial option) or a low-value food (selfish option) to a recipient (a low-ranking adult male) using a pulling tool. In some trials, the actor monkey received a high-value food regardless of his choice, and in others he received a low-value food regardless of his choice. Comparing the number of times each actor selected the prosocial option when a partner was present to retrieve food rewards (equivalent to *prosocial test sessions* in this study) versus when no partner was present (equivalent to *empty control sessions* in this study), we found that JM selected the prosocial option significantly more often only when she received a low-value food (*p* = 0.002). That is, JM preferred to deliver a high-value reward to the adjacent chamber more often when a recipient was present than when no recipient was present on those trials in which JM herself received a low-value reward, but not when she received a high-value reward.

Results from both studies are summarized in Table 1. Notably, whereas in the current study JM never exhibited prosocial behavior, in a previous study she did so. In contrast, NN behaved prosocially in the current study but not in Lakshminarayanan and Santos [15]. Finally, FL did not behave prosocially in either of the studies. It is also worth noting that despite the fact that JM and NN both exhibited prosocial preferences in two different studies in some conditions, both also failed to exhibit significant prosocial behavior within those same studies under slightly different conditions.

**Table 1:** Comparison of Actors’ Behavior across Studies.

Study	Actor’s payoff regardless of choice	FL	JM	NN
Current study	Food reward	ns	ns	*
	No food reward	ns	ns	+
Lakshminarayanan and Santos (2008)	High-value food	ns	ns	ns
	Low-value food	ns	*	ns

*^+^ p* < 0.06, ^*^ *p* < 0.05; significance levels indicate whether the actor selected the prosocial option more often than expected based on that actor’s performance when no recipient was present.

## 4. Discussion

The goal of this study was to examine whether capuchin monkeys would exhibit prosocial behavior using a novel touchscreen task. Although previous studies have documented prosocial preferences in capuchins [12,15,20], the same species showed little evidence of such preferences in our task. When presented with the choice between 1/1 and 1/0, capuchin actors did not choose the prosocial option at levels significantly above chance. More importantly, actors did not deliver a food reward to the blue cup more often when either the recipient or the actor had access to the blue cup compared to when no one did. When presented with the choice between 0/1 and 0/0, actors chose the prosocial option at levels significantly above chance in all conditions but again selected the prosocial option equally often across the three conditions.

Our results might at first glance seem to contradict those of previous studies reporting that capuchins demonstrate prosocial preferences, but it is important to consider exactly what subjects understood about our task before making such a conclusion. Although it is possible that capuchins did not understand the specific stimuli used in this study, their responses to the two pairs of stimuli in which one option was always preferable regardless of condition type show that this was not the case. For both pairs of stimuli in which one option was always superior to the other (*i.e.*, 1/1 *vs.* 0/1 and 1/0 *vs.* 0/0), capuchins chose the optimal (*i.e.*, food maximizing) option at levels significantly greater than chance across all conditions. Indeed, proposers were quite adept at choosing among these pairs, as a group selecting the choice that maximized their own reward outcome on over 87% of all trials.

These results present a puzzle in that proposers clearly did understand something about the reward outcomes of the stimuli, but failed to behave differently when their prosociality was tested in the three conditions. This was expected for two of the four pairs of stimuli, but we anticipated that when actors were presented with the two pairs in which there was a prosocial option and a selfish option, they would at least choose the prosocial option more often when they themselves would benefit by doing so (that is, in the *selfish control condition*). That capuchins did not do so suggests that they were not necessarily attending to the contingencies of the different conditions. Although our training procedure was specifically designed to familiarize actors with the fact that reward outcomes varied across the different conditions and mirrored training previously done in other capuchin prosociality studies, it is possible that actors simply learned to associate each stimulus with, on average, a certain number of food rewards. Consequently, when given a choice between two pairs of stimuli, the strength of actors’ preference for one stimulus over another may merely reflect that probability of receiving a certain number of food rewards generally and may be insensitive to the contingencies specific to a given condition. The group data therefore highlight the importance of including a condition designed to probe subjects’ understanding of the task. In this case, subjects’ behavior in the *selfish control condition* cautions against concluding that capuchins do not have prosocial preferences. In addition, our results highlight the benefit of assessing subjects’ understanding of the testing paradigm prior to, as well as parallel with, the testing stage. It’s unclear precisely why subjects failed to learn the contingencies of our task, but it is possible that the complexity of the touchscreen apparatus played some role. Other studies have used (arguably) less complex methods, such as token exchange paradigms [12] or pulling a physical plank or drawer containing food rewards [15,20]. In such cases, the actor can often see the causal sequences between his own choice and the resulting food distribution, which may facilitate task understanding.

Despite the failure of the group to display prosocial behavior, an examination of each individual actor’s data revealed that when given the choice between 1/1 and 1/0 one of the four subjects, NN, did choose the prosocial option more often when a recipient was able to retrieve food rewards delivered to the blue cup compared to when no one had access to the blue cup. NN also showed a trend towards doing so when presented with 0/1 and 0/0. Although it is tempting to speculate as to why only NN demonstrated prosociality in this particular context, the fact that none of the subjects clearly understood the task makes such speculation difficult since other subjects may have chosen prosocially had they understood all the reward contingencies. NN was also the only subjects to be presented with the *prosocial test session* last in each of the three testing sets, and so we cannot completely rule out that possibility that order effects played some role in subjects’ performance. Furthermore, although NN’s behavior may appear to be prosocially motivated, it is unclear why he did not choose the prosocial option more often in the *selfish control condition*. Thus, while we think it is possible that NN’s behavior reflects true prosocial motivation, it is unclear as to whether this is the most parsimonious explanation of the data.

Similarly, although an initial goal of this study was to explore whether some individuals might be consistently more prosocial than others by comparing subjects’ performance in the current study and Lakshminarayanan and Santos [15], variation in subjects’ performance across the two studies seems likely to reflect differences in task understanding rather than prosocial motivation per se. Nevertheless, we hope that future studies will continue to explore individual variation in prosociality by testing the same individuals across a range of tasks. Although group data is obviously informative, looking at prosocial preferences at the level of the individual can also provide important insights. For example, examining each individual’s performance reveals what proportion of subjects actually demonstrated an understanding of the task. Obviously if subjects do not understand the task, we cannot draw any conclusions about whether they do or do not show prosocial preferences. Individual level analyses are necessary to determine whether significant group preferences for prosocial outcomes reflect the behavior of most individuals within that group or whether a few subjects may be largely driving the effect. Such data can help inform debates about the degree to which prosocial behaviors, when observed, are species typical and truly indicative of a robust preference to help others. It is also possible that some subjects that exhibit prosocial behavior do not necessarily seem to understand all aspects of the task (as in our study). In these cases, researchers will want to treat results with caution.

Finally, we add that when exploring inter- and intra-individual consistency in performance, it may be particularly valuable to include both tasks involving donating food to other group members as well as tasks involving other measures of prosociality as there is some doubt as to whether food donation tasks are the best way to assess prosociality in other species. For example, although studies testing prosociality in chimpanzees using food donation tasks have produced mostly negative results [9,14,18], other studies have found that chimpanzees are willing to help others in instrumental helping tasks [3,4]. This has led to the suggestion that visible food rewards may in some cases inhibit prosociality, perhaps because they elicit competitive responses from subjects [13,31]. Consistent with this, in the one study that found strong evidence of prosociality in a chimpanzees study using a food donation task, food was concealed in paper during the choice phase of the experiment [13]. Understanding how individuals behave across a range of differently structured prosocial tasks will therefore allow researchers to not only learn more about inter-individual variability in prosociality, but also allow us to better assess how different species may construe these tasks and ultimately lead to the development of better testing paradigms.

## 5. Conclusions

Exploring prosociality in nonhuman primates has the potential to provide important insights into the evolutionary origins of human altruism. Although studies testing other-regarding preferences in primates have been the subject of much interest in recent years, these studies have produced a set of results that remain difficult to explain under a unified theoretical framework. We believe that one potentially informative line of inquiry involves testing the stability of prosocial behaviors, both at the species and individual level. We attempted to do just this in the present study. Unfortunately, subjects’ limited understanding of our task prevents us from drawing any definite conclusions based on our results. Nevertheless, we hope that future research will continue to address this question.
